# Exploring the Relationship between Gray and White Matter in Healthy Adults: A Hybrid Research of Cortical Reconstruction and Tractography

**DOI:** 10.1155/2021/6628506

**Published:** 2021-03-11

**Authors:** Yongxiang Zhao, Qianqian Li, Jiachen Du, Hongjian He, Peipeng Liang, Jie Lu, Kuncheng Li

**Affiliations:** ^1^Department of Radiology, Xuanwu Hospital, Capital Medical University, Beijing 100053, China; ^2^Key Laboratory of Magnetic Resonance Imaging and Brain Informatics, Beijing 100053, China; ^3^Center for Brain Imaging Science and Technology, Zhejiang University, Hangzhou 310007, China; ^4^School of Psychology, Capital Normal University, Beijing 100037, China

## Abstract

The gray matter (GM) and white matter (WM) are structurally and functionally related in the human brain. Among the numerous neuroimaging studies, yet only a few have investigated these two structures in the same sample. So, there is limited and inconsistent information about how they are correlated in the brain of healthy adults. In this study, we combined cortical reconstruction with diffusion spectrum imaging (DSI) tractography to investigate the relationship between cortical morphology and microstructural properties of major WM tracts in 163 healthy young adults. The results showed that cortical thickness (CTh) was positively correlated with the coherent tract-wise fractional anisotropy (FA) value, and the correlation was stronger in the dorsal areas than in the ventral areas. For other diffusion parameters, CTh was positively correlated with axial diffusivity (AD) of coherent fibers in the frontal areas and negatively correlated with radial diffusivity (RD) of coherent fibers in the dorsal areas. These findings suggest that the correlation between GM and WM is inhomogeneity and could be interpreted with different mechanisms in different brain regions. We hope our research could provide new insights into the studies of diseases in which the GM and WM are both affected.

## 1. Introduction

The human brain is a complex integrity consists of gray matter (GM) and white matter (WM). The cooperation of GM and WM is essential for basic and senior brain functions. Over the last two decades, the fast advancing neuroimaging techniques facilitated noninvasive investigations of these two brain structures [1, 2]. Given their different microstructural characteristics, different magnetic resonance imaging (MRI) modalities were often used to assess these two structures. T1-weighted image in the structural MRI (sMRI) offers a clear contrast between the GM and WM; so, automatic brain segmentation and cortical reconstruction were used to acquire the anatomical information of the GM cortex [3]. And the diffusion MRI (dMRI) can measure the Brownian motion of water molecules in vivo; so, it was used to trace the WM fiber tracts and quantitively analyze WM microstructural status [4]. Due to the divergence in MRI modality selection, only a few studies have simultaneously investigated the GM and WM in the same sample. These studies provide the primary information about the relationship between these two structures in the group level [5, 6].

In some diseases, previous multimodal studies have shown that both the GM and WM undergo changes, and the changes are correlated in certain regions [7–9]. For the healthy people, some investigators have tested the relationship between GM and WM but their findings were inconsistent. For instance, Kochunov et al. reported a positive relationship between cortical thickness (CTh) and WM fractional anisotropy (FA) value of the whole brain [5], while Tamnes et al. reported that CTh was negatively correlated with underlying WM FA values in most areas [6]. And some studies also showed spatial discrepancy of the GM-WM relationship. For instance, Wu et al. found that the CTh and adjacent superficial WM FA were positively correlated in sensorimotor, visual, and auditory areas, but negatively correlated in high-order functional areas such as the prefrontal cortex [10]. Due to the limited number of studies and the inconsistent conclusions, there is still no consensus about the exact relationship between GM and WM in the brain of healthy adults.

In this study, we aimed to investigate the relationship between cortical morphology and microstructural status of major WM tracts. High quality sMRI and dMRI image data was collected from a multicenter project of healthy adults. In image processing, we firstly performed automatic surface-based cortical reconstruction on the T1 data and then used manual ROI drawing tractography performed on the diffusion spectrum imaging (DSI) data to obtain the diffusion parameters of major association and commissural fibers. Finally, we used the general linear model (GLM) to test the correlation between the tract-wise WM properties and vertex-wise cortical morphological indices. Referring to the previous studies, we hypothesized that (1) the CTh and coherent tract-wise FA values are positively correlated. And the correlation is stronger in the dorsal areas. (2) Joint measurement of tract-wise axial diffusivity (AD) and radial diffusivity (RD) indices may further help to explain the mechanism underlying the GM-WM relationship. (3) The correlation with major WM parameters is mainly related to CTh rather than the cortical surface area (CSA).

## 2. Material and Methods

### 2.1. Participants

Our research data was acquired from a multicenter brain research project named China-brain-atlas (http://www.cbatlas.org). In this project, we advertised to hire more than 1,600 healthy adults into 11 medical centers in China. All participants had taken a set of physical exams and cognitive scale tests beforehand. The participants with (1) abnormality in cognitive test, (2) physical illness, (3) nervous system disorder, and (4) MRI contraindications were excluded. The remaining participants took the MRI exam. And after MRI scan, those of poor images quality or with brain lesion on structural images were also excluded. Finally, 998 participants were included in the research project. All the participants were right-handed native Chinese speakers. The MRI scans were done between Oct. 2016 and Oct. 2018.

For this study, we selected participants ≤ 35 years old in five large centers, and 163 participants were finally included [with age range of 19-35 years, average age 27.1 ± 4.2 years, 84 females (26.7 ± 4.1)/79 males (27.4 ± 4.3)]. This study was approved by the ethics committee of Xuanwu Hospital, and all the participants have signed informed consent before commencement of the study.

### 2.2. Image Acquisition

The scanning hardware and software were uniform in all imaging centers. The MRI scanners were 3 T MAGNETOM Prisma (Siemens AG, Erlangen, Germany) with the XR 80/200 gradient system. And the same model 64-channel head coils were used. The suited software was the Syngo MR VD13D. A Dot Engine for autoalignment was used to minimize the registration error between subjects.

The T1-weighted images were acquired using a 3D Magnetization Prepared 2 Rapid Acquisition Gradient Echoes (MP2RAGE) sequence (TR/TE = 5 s/2.9 ms, TI = 700, 2500 ms, FOV = 25.6 × 25.6 cm, voxel size = 1.2 × 1 × 1 mm^3^). Diffusion-weighted images were acquired using a simultaneous multislice (SMS) echo planner imaging (EPI) prototype sequence (TR/TE = 5.4 s/71 ms, FOV = 22 × 22 cm, slice number = 93, voxel size = 1.5 × 1.5 × 1.5 mm^3^, bandwidth = 1712 Hz/Px, iPAT factor = 2, and SMS factor = 3). The *b* values were 1000, 2000, and 3000 s/mm^2^. Each shell containing 30 vectors with uniform angular coverage was generated from a multiple q-space sampling tool, with additional 6 b0 volumes. The data was collected in two opposite directions (AP-PA) for eliminating susceptibility induced distortions. A previous study by our group was conducted to confirm the reproducibility of this intercenter dMRI data acquisition protocol [11].

### 2.3. Cortical Reconstruction

Automatic surface-based cortical reconstruction for the T1-MP2RAGE images was performed with the Freesurfer v6.0 routine pipeline. Detailed descriptions of the cortical reconstruction procedure could be found on the website (http://surfer.nmr.mgh.harvard.edu/fswiki/FreeSurferMethodsCitation) [3]. In brief, after preprocessing, intensity and continuity information were used to reconstruction the GM-WM boundary. Then, the cortical pial surface and gray/white interface were tessellated into triangles, and the cortical mantle was inflated and registered to a spherical atlas. A total of 163,842 vertexes were assigned to each hemisphere. The CTh was defined as the closest distance between the gray/white interface to the pial surface at each vertex. The CSA was computed as a summation of each triangle near a vertex. And the cortical volume (CVo) is a mixture of the CTh and CSA. Those surface-based maps were smoothed using a Gaussian kernel with a full width half maximum (FWHM) of 15 mm. The estimated total intracranial volume (eTIV) offered by Freesurfer was also recorded.

### 2.4. Diffusion Data Preprocessing and Tractography

The diffusion data preprocessing and fiber tracking procedure were achieved using the DSI-Studio software (http://dsi-studio.labsolver.org/Manual). This software uses the generalized q-sampling imaging (GQI) algorithm to generate the spin distribution function (SDF) map for deterministic tractography [12]. The DSI data preprocessing procedures include (1) correct for susceptibility-induced distortions using reversed phase-encode (AP-PA) image data, (2) use eddy correction to reduce the eddy currents and subject motion, (3) set a brain mask semiautomatically to reduce the time consumption of fiber tracking, (4) use GQI to reconstruct the SDF and quantitative anisotropy (QA) map with fiber length ratio at 1.1, and (5) fit the diffusion tensor model to calculate the tensor eigenvalue map of FA, AD, RD, and mean diffusivity (MD).

**Figure 1:**
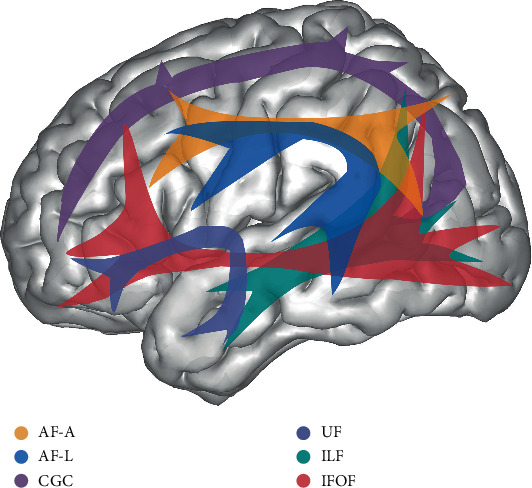
The association fibers. Orange: the arcuate fasciculus anterior segment (AF-A), blue: the arcuate fasciculus long segment (AF-L), purple: cingulum bundle-cingulate gyrus (CGC), blue purple: uncinate fasciculus (UF), green: inferior longitudinal fasciculus (ILF), and red: inferior frontal-occipital fasciculus (IFOF).

**Figure 2:**
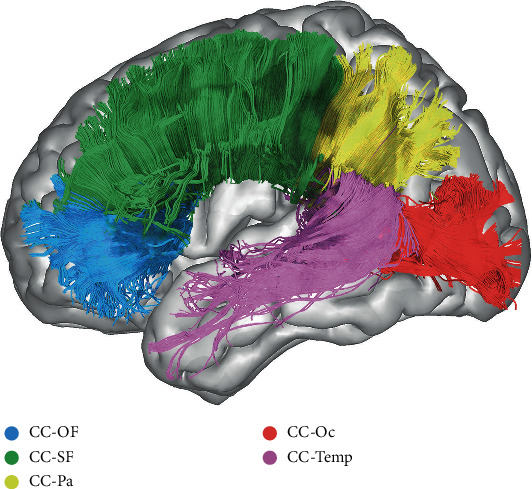
The corpus callosum (CC). Blue: the orbital frontal part of CC (CC-OF), green: the superior frontal part of CC (CC-SF), yellow: the parietal part of CC (CC-Pa), red: the occipital part of CC (CC-Oc), and pink: the temporal part of CC (CC-Temp).

Using the SDF map, a whole brain tractography was first established. The tracking parameters were set referring to the manual and adjusted for our data for better valid connection ratio: the anisotropy threshold as default, different tracking thresholds at 0.2, angular threshold at 50°, a step size of 0.5, smoothing threshold at 0.2, fiber length range between 30 and 240 mm, and tractography termination of 350,000 seeds. We used ROI-based delineation to acquire the positional information of major fiber tracts. In this method, two or more ROIs were manually delineated around the fiber stem on the principal direction color map. The streamlines penetrating all the ROIs were assigned as the specific fiber [13]. In addition, regions of avoidance (ROAs) were used to exclude the invalid streamlines. The ROI drawing protocol (details in Supplementary Figure 2 and 3) in this study was an integration and modification of the previous studies [14–17]. The definitions of the association fibers and commissure fibers we traced are listed as follows (Figures 1 and 2).

The perisylvian language pathway, also known as the arcuate fasciculus (AF), is composed of three bundles of fibers running around the lateral fissure: the anterior segment (AF-A), long segment (AF-L), and posterior (AF-P) segment [18]. In this study, we traced bilateral anterior and long segments of AF. The AF-A, also called superior longitudinal fasciculus (SLF) II, connects the ventrolateral frontal cortex with the inferior parietal cortex. And the AF-L connects the frontal cortex with the superior and middle temporal cortex.

The cingulum bundle is a ring-shaped fiber tract that runs around the corpus callosum, with fibers joining and leaving it in different lobes [19]. It includes two separate parts, the upper part mainly runs in the cingulate gyrus (CGC), and the lower part runs near the hippocampus (CGH). For simplification, we only traced the CGC in this study.

The ventral language pathway includes a series of fiber tracts running in the ventral part of the brain such as the uncinate fasciculus (UF), the inferior frontal-occipital fasciculus (IFOF), and the inferior longitudinal fasciculus (ILF) [17, 20]. The UF is a U-shaped fiber bundle connecting the inferior frontal with the anterior temporal areas. The IFOF, passing through the extreme capsule (EmC), connects the frontal lobe with the occipital and parietal lobes. The ILF connects the temporal lobe with the occipital and parietal lobes.

The corpus callosum (CC) is the largest commissure fiber which connects the bilateral brain hemisphere within the symmetric area. According to previous studies [21, 22], we divided the CC into five parts based on its connection area, including the orbital frontal area (CC-OF), the anterior and superior frontal area (CC-SF), the parietal lobe (CC-Pa), the occipital lobe (CC-Oc), and the temporal lobe (CC-Temp).

After tractography, we recorded the FA, MD, AD, RD, and QA values of each tract, and these eigenvalues were averaged across all voxels in the tract. The number of fiber streamlines (NoF) in each tract was also recorded. The manual-ROI tractography was conducted by one radiologist (YZ) with >5 years of experience of dMRI processing. In order to verify the reproducibility of our fiber tracking pipeline, we chose 10 subjects to test the interrater reliability between two radiologists (YZ and QL) in a pilot study.

### 2.5. Statistical Analysis

To test the relationship between tract-wise diffusion parameters and cortical morphological indices, we fit a GLM at each vertex across the cortical mantle to explain the data from all subjects in the study. The vertex-wise surface-based statistical analyses were performed with the QDEC toolbox of Freesurfer (https://surfer.nmr.mgh.harvard.edu/fswiki/FsTutorial/GroupAnalysis). We calculated the correlation between the FA, MD, AD, RD, NoF, and QA of each fiber bundle with vertex-wise cortical indices (CTh, CVo, and CSA) (the association fibers' dMRI properties with its ipsilateral cortex and the commissure fibers' dMRI properties with bilateral cortex). We controlled for the effect of age in all analyses and further controlled for the eTIV in the analysis with CSA and CVo [23]. A cluster-wise multicomparison correction was done using Monte Carlo simulation with a threshold *p* < 0.05 (certain results are shown in Supplementary material). Given the widespread and matching correlations, results were also presented with original *p* value maps (with a threshold of *p* < 0.01) to better visualize the spatial pattern of GM-WM correlation. This visualization process was conducted with the SurfStat toolbox (http://www.math.mcgill.ca/keith/surfstat/).

## 3. Results

### 3.1. The Outcome of Tractography

In the pilot study, we tested the interobserver reliability between two radiologists for 10 randomly selected subjects. For the major tracts, the interclass correlation coefficient (ICC) for each index was NoF: 0.915, FA: 0.979, MD: 0.974, AD: 0.940, RD: 0.978, and QA: 0.944. Then, for the whole dataset, we successfully traced all designated fiber bundles for each subject. The detailed parameters of tract-wise WM indices are listed in Supplementary Table 1. As expected, for each tract, no clear linear correlation between the tract-wise FA value and age was found.

### 3.2. The Relationship between Tract-Wise FA Value and Cortical Morphology

**Figure 3:**
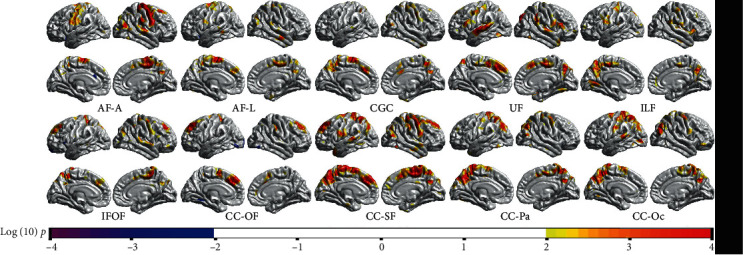
The correlation between tract-wise FA values and vertex-wise CTh (with a threshold of *p* < 0.01). For most tracts we traced, there were positive correlations between the FA value and coherent CTh. The correlation was stronger in the dorsal areas (e.g., the superior-frontal gyrus, the precentral gyrus, the parietal lobe, and the superior temporal gyrus with coherent fibers as AF-A, CGC, CC-SF, and CC-Pa).

The results of the GLM test for the correlations between tract-wise FA values and vertex-wise CTh are shown in Figure 3. For most fiber bundles we traced, there were positive correlations between tract-wise FA values and CTh of corresponding areas. The correlations were stronger in the dorsal areas (e.g., the superior frontal gyrus, precentral and postcentral gyrus, superior temporal gyrus, and precuneus with coherent fibers as AF-A, CGC, CC-SF, and CC-Pa), but weaker in the ventral areas (e.g., the middle and inferior frontal gyrus, inferior temporal gyrus, and the occipital lobe with coherent fibers). Most of these correlations survived the Monte Carlo simulation correction (Supplementary Figure 4). No prominent negative correlation between tract-wise FA and CTh was found.

There were moderate positive correlations between most tract-wise FA values with corresponding CVo. The spatial pattern looked similar to that of the FA-CTh correlations but the correlation strength was weaker (Supplementary Figure 5). No prominent correlation between tract-wise FA value and CSA was found.

### 3.3. The Relationship between Tract-Wise AD/RD and Cortical Morphology

**Figure 4:**
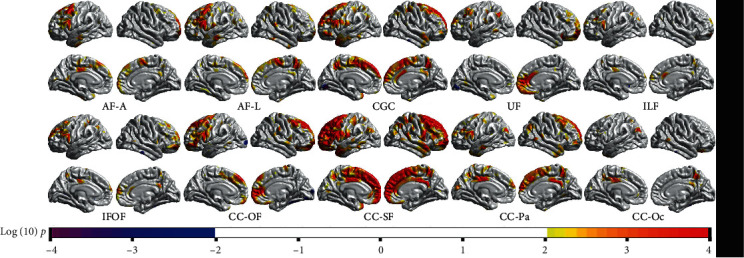
The correlation between tract-wise AD values and vertex-wise CTh (with a threshold of *p* < 0.01). For the fibers that had connection to the frontal cortex (e.g., AF-A, CGC, CC-OF, and CC-SF), there were positive correlations between their AD value and corresponding CTh. In posterior areas, no prominent correlation between tract-wise AD value and CTh was found.

The correlations between tract-wise AD and CTh are shown in Figure 4. For the fibers with connections to the frontal area, such as the CGC, IFOF, CC-OF, and CC-SF, there were positive correlations between their AD and corresponding CTh (e.g., superior and middle frontal lobe and frontal pole). Most of these correlations survived the Monte Carlo simulation correction (Supplementary Figure 6). And for the fibers running in the temporal and occipital lobes such as the ILF and CC-Oc, there was no prominent correlation between their AD and corresponding CTh. In addition, the correlations between CVo and tract-wise AD were similar but much weaker compared with the CTh-AD correlations (Supplementary Figure 7). No significant correlation was found between CSA and tract-wise AD.

**Figure 5:**
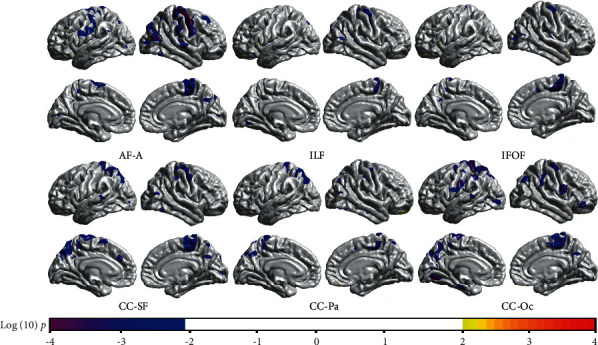
The correlation between tract-wise RD values and vertex-wise CTh (with a threshold of *p* < 0.01). For the tracts that had connections to the dorsal areas (e.g., AF-A, CC-SF, CC-Pa, and CC-Oc), there were negative correlations between tract-wise RD and corresponding CTh. The results of fibers which showed no significant correlation with CTh are not given.

The correlations between tract-wise RD and CTh are shown in Figure 5. For the fiber tracts (e.g., AF-A, CC-SF, CC-Pa, and CC-Oc) with connections to the dorsal cortices (e.g., the precentral gyrus and the parietal lobe), there were negative correlations between the RD values and corresponding CTh. Most of these correlations survived the Monte Carlo simulation correction (Supplementary Figure 8). While in the anterior and ventral areas, no prominent correlation was found. There was no significant correlation between tract-wise RD and CVo or CSA.

### 3.4. Other Correlations

There were positive correlations between the CTh and the MD of CGC, UF, IFOF, and CC-OF in the frontal areas. The spatial pattern looked similar to that of the CTh-AD correlations but the strength was weaker. And tiny negative correlations could be found between CTh and MD of AF-A and UF in the dorsal areas (Supplementary Figure 9). No significant correlation between the tract-wise MD and CSA or CVo was found.

We also tested the correlation between tract-wise QA/NoF and cortical morphological indices, but no significant result was found.

## 4. Discussion

The aim of this study was to investigate the relationship between cortical morphology and microstructural properties of major WM tracts. The results showed that the CTh was positively correlated with FA of coherent WM tracts. As discussed in earlier neuroimaging studies, the CTh is a reflection of the neuron numbers in the cortical column [24], and the FA is predominantly related to the WM integrity [25]. The positive correlations indicate that more neurocytes in the cortical column are corresponding to higher integrity of coherent WM tracts.

In the previous multimodal studies, some investigators had tested the correlation between cortical morphology and WM microstructural properties, but their findings were inconsistent. For the whole brain, Kochunov et al. reported a positive correlation between the GM CTh and WM FA value in 1031 healthy people aged 11–90 years [5]. While in some studies of children and adolescents, Tamnes et al. showed that the CTh was negatively correlated with adjacent WM FA in most areas (negative in 22 and positive in 4 of total 33 regions) [6]. And Wu et al. reported a negative correlation between CTh and underlying superficial WM FA value in the prefrontal and parietal areas [10]. Giorgio et al. also reported a strong negative correlation between GM density and WM FA in right frontal lobe [26]. The negative correlations reported in these studies differ from our findings. We noticed that in these studies, the majority of participants were adolescents. The GM and WM follow chronologically different development-degeneration trajectories [27]. In the adolescents, there are synchronous cortical thinning and coherent WM microstructural maturation [28]. Thus, younger subjects correspond to thicker cortex but lower WM FA values, and older subjects correspond to thinner cortex but higher WM FA values, which may result in the negative correlation between CTh and FA [29]. As seen in the supplementary materials of Tamnes' study, after controlling for age, the negative correlations were significantly weakened [6]. And in the studies of adults, when the GM and WM both undergo degeneration process, the primary correlation between GM and WM was found to be positive [5, 30, 31]. In this study, we chose an age range in which the FA increment had nearly plateaued and included age as a covariate. The result that alleviated the effect of age may better reflect the intrinsic correlation between GM and WM.

In this study, we also found that the correlation between CTh and tract-wise FA was stronger in the dorsal areas than in the ventral areas. We reviewed previous studies and found a similar trend. For instance, Wu et al. found positive correlations between superficial WM FA and the corresponding CTh in the unimodal areas (e.g., sensorimotor cortex and visual and auditory cortex) and negative correlations in the multimodal areas (e.g., dorsal lateral prefrontal cortex) [10]. Fjell et al. found positively correlations between CTh and WM FA in bilateral precentral gyrus, lingual gyrus, and precuneus [30]. Hoagey et al. also reported that the FA of the AF-A, CGC and posterior projections of ILF and IFOF, CC-SF, and CC-Pa showed greater covariance with GM CTh [32]. Moreover, some studies showed that CTh and FA were negatively correlated in the middle frontal gyrus [6, 29]. These studies suggest that the correlation between CTh and FA is stronger in the dorsal areas but weaker or negative in the ventral areas. A plausible explanation is that the dorsal cortices (e.g., the sensorimotor, visual, and auditory cortex) is simpler in function and has more homogenous neuronal connectivity [10]. The increment of neuron number in the cortex will directly lead to more coherent axon or better myelination of underlying fibers. While in the high-order functional areas (e.g., middle and inferior frontal gyrus and inferior temporal gyrus), the fiber connections are more complicated. So, there are more crossing fibers in these areas [33–35]. As reported in earlier studies, in fibers crossing areas, sparing rather than dense fibers is correlated with higher FA value [36]. So, in these areas, thicker cortex corresponding to more dense fibers may not necessarily lead to higher FA value.

The diffusion tensor model offers multiple parameters reflecting different aspects of WM microstructure. For instance, the AD representing diffusivity along the neuronal axons is sensitive to the axonal coherence, density, and integrity. Lower AD value could be found in axonal injury, and higher AD value is often related to more dense and intact axons [37]. The RD representing diffusivity perpendicular to the axons is sensitive to myelination status, and lower RD value often means better axonal myelination [38, 39]. In this study, there were positive correlations between the CTh and AD of coherent fibers in the frontal areas. And there were negative correlations between the CTh and coherent tract-wise RD value in the dorsal or posterior areas. And in the previous studies, Tamnes et al. showed that the CTh and coherent WM AD value was positively correlated in the frontal lobe [6]. Wu et al. found RD and FA synchronously change in bilateral motor sensory cortices and superior temporal auditory cortex [10]. Based on these findings, we consider that the coordination between GM and WM could be interpreted by different mechanisms in different brain regions. In the frontal areas, also recognized as high-order functional areas, the CTh representing number of neurocytes in the cortical column is positively correlated with axonal density of coherent WM. While in the dorsal areas, which are often related to basic functions such as sensorimotor, visual, and auditory, the CTh is more closely related to the myelination level of underlying WM tracts.

The surface-based cortical reconstruction offers two detailed indices that define the CVo: CTh and CSA. Earlier studies have suggested that using these indices separately will better reflect the intrinsic features of the cortex [40]. So, we tested the correlations between WM parameters and CTh/CSA, respectively, and found that tract-wise WM parameters showed significant correlations with the CTh rather than CSA. As shown in the previous studies, the CSA and WM volume were positively correlated [41]. When the CSA changes, the axonal density may remain stable due to simultaneous WM volume change. This may cause the absent of correlation between tract-wise WM parameters and corresponding CSA. This study also showed that the correlations between CVo and FA were similar to that of CTh-FA but the strength was weaker. As the CVo is a combination of CTh and CSA, its interaction with WM properties could be regarded as a reflection of the CTh-WM relationship [42]. Considering these cases, in the research concerning the coordination between GM and WM, the CTh maybe a better choice than the CVo to reflect cortical morphological properties. And using CSA alone may overlook the correlation between GM and WM [32].

In some diseases, previous multimodal studies have shown that both the GM and WM undergo pathological changes. For instance, in multiple sclerosis (MS) and spinal cord injury (SCI), the primary pathology process such as the demyelination or injury of the corticospinal tract was often followed by sensorimotor cortical thinning [43, 44]. And in some psychological and neurodegenerative diseases, such as schizophrenia, mild cognitive impairment (MCI), and Alzheimer's disease, cortical thinning and relevant WM degeneration could both be found [7, 45, 46]. Some studies further showed that the changes are correlated in certain regions. For instance, in patients of Alzheimer's disease, Sydykova et al. found that FA decline in the corpus callosum was significantly correlated with cortical atrophy of corresponding areas [9]. And in schizophrenia patients, Liu et al. found that lower FA in ILF and IFOF was associated with adjacent orbitofrontal and temporal cortical thinning [47].

The combination of sMRI and dMRI can improve the diagnostic accuracy for certain diseases and improve the effectiveness of the lesion localization. Moreover, some studies found no correlation between GM and WM in their healthy control group [9, 43, 46], and given the results of our study, certain negative results may be due to a bias of the small sample size.

This study has several limitations. First, the result of tractography could be affected by noise, artifacts, and other limitations of the diffusion model. The fibers that shared the path or had more intersections with others could be interfered by the neighboring fibers; so, their interaction with the cortex maybe biased. Second, the interaction between GM and WM maybe more complex concerning the age effect. To simplify the design of this study, we selected an age group when the WM maturation had plateaued and corrected for age as covariance. But through this practice, we may be unable to systematically show the GM-WM correlation patterns in different age groups, especially in the elderly who are susceptible to neurodegenerative diseases. We hope further studies concerning larger age span or using longitudinal design could help better know the spatial and temporal patterns of the GM-WM relationship.

## 5. Conclusion

In this study, we combined cortical reconstruction with DSI tractography to test the correlations between the GM and WM. The result showed that CTh was positively correlated with the coherent tract-wise FA value especially in the dorsal areas. Using other diffusion parameters, we found that the CTh was positively correlated with coherent axonal density in the frontal areas and coherent axonal myelination of in the dorsal areas. These findings suggested that the correlation strength between GM and WM and underlying mechanism were inhomogeneous in different brain areas. We hope this study could help to better understand the coordination of different brain structures and facilitate future multimodal studies of brain diseases.

## Data Availability

The data used to support the findings of this study are available from the corresponding author upon request.

## Supplementary Materials

Supplementary Materials Supplementary Figure 1: data analysis pipeline. Supplementary Figures 2 and 3: ROI drawing protocol for tractography. Supplementary Figures 4-9: the correlations between GM and WM after correction. Supplementary Table 1: major results of tractography.
