# Supplementary material for: Exploring the Relationship between Gray and White Matter in Healthy Adults: A Hybrid Research of Cortical Reconstruction and Tractography
Source: Biomed Res Int. 2021 Mar 11;2021:6628506. doi: 10.1155/2021/6628506 (PMC7979294; doi:10.1155/2021/6628506)
Supplement: Supplementary Materials — Supplementary Figure 1: data analysis pipeline. Supplementary Figures 2 and 3: ROI drawing protocol for tractography. Supplementary Figures 4-9: the correlations between GM and WM after correction. Supplementary Table 1: major results of tractography. [file 6628506.f1.docx]

**SUPPLEMENTARY MATERIAL**

**Exploring the relationship between gray matter cortex and** **white matter tracts in healthy adults:a hybrid research of cortical reconstruction and tractography**

Yongxiang Zhao*^1,2^*, Qianqian Li*^1,2^*, Jiachen Du*^1,2^*, Hongjian He*^3^*, Peipeng Liang*^4^*, Jie Lu*^1,2^*, Kuncheng Li*^1,2*^*

*1. Department of Radiology, Xuanwu Hospital, Capital Medical University, Beijing, China*

*2. Key Laboratory of Magnetic Resonance Imaging and Brain Informatics, Beijing, China*

*3. Center for Brain Imaging Science and Technology, Zhejiang University, Hangzhou, China*

*4. School of Psychology, Capital Normal University, Beijing, China*

*** Corresponding author:** cjr.likuncheng@vip.163.com

1. **Data analysis pipeline**


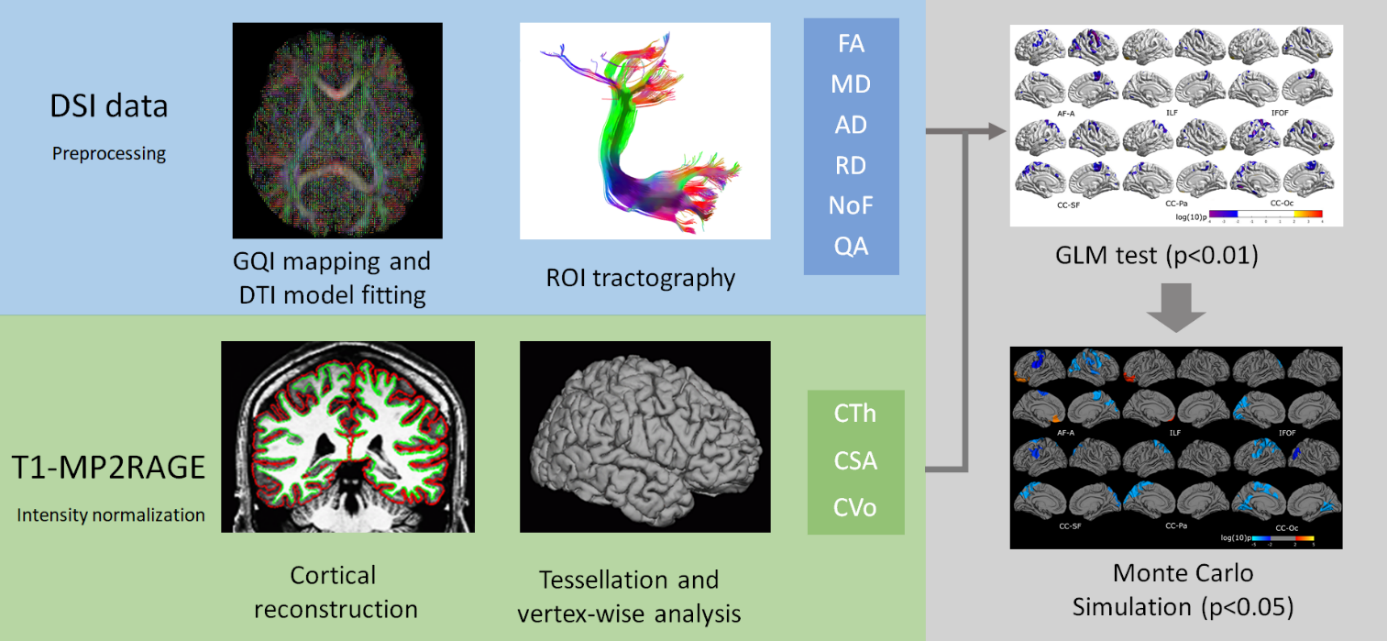


**Supplementary Fig. 1.** Data analysis pipeline

1. **ROI drawing protocol for tractography**


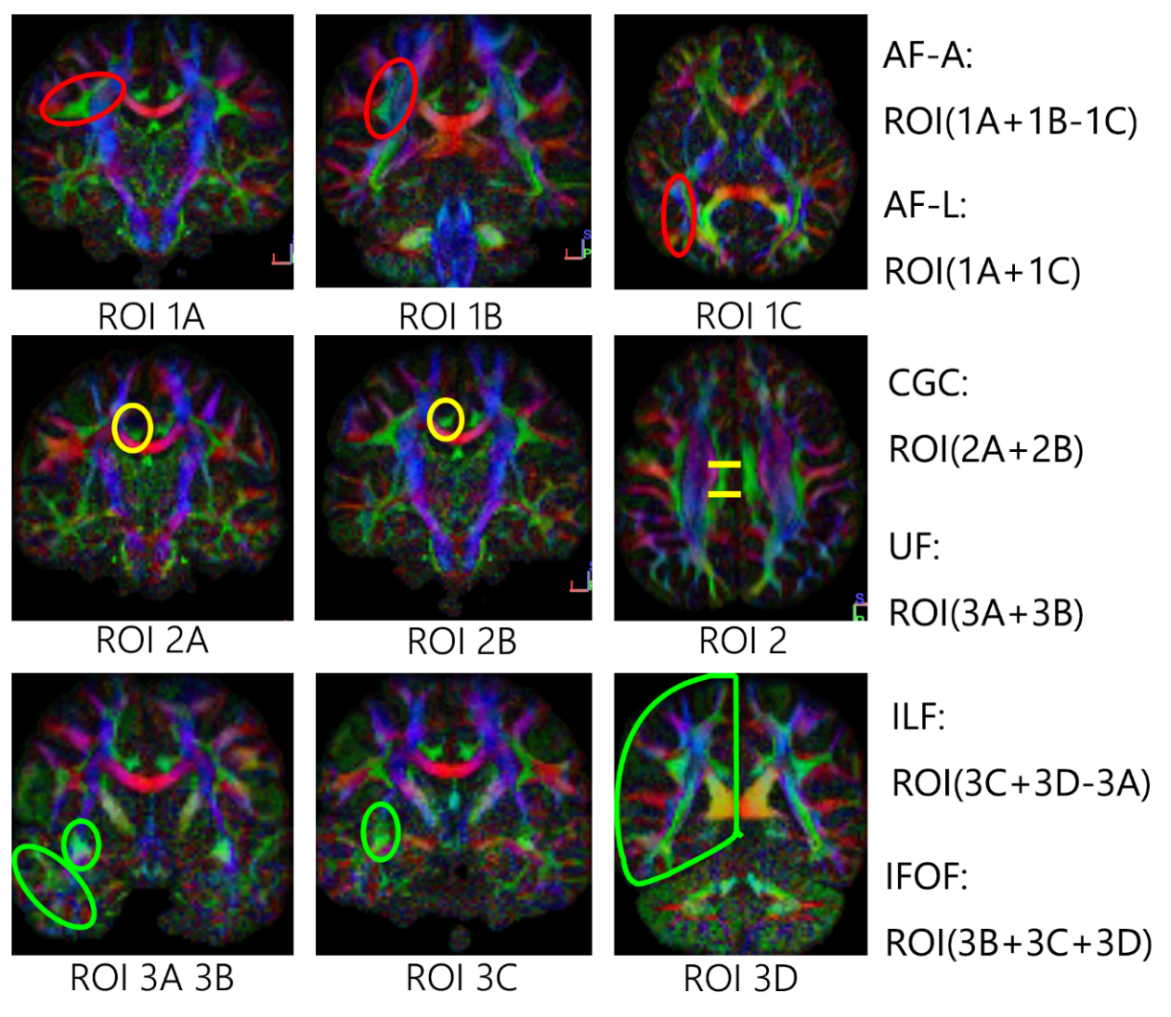


**Supplementary Fig. 2.** The ROIs placement for association fibers

The arcuate fasciculus anterior and long segment (AF-A/ AF-L): ROI 1A was drawn on the coronal slice at the middle of posterior limb around the green triangle, ROI 1B was drawn on the coronal slice at the splenium of corpus callosum around the green stripe, ROI 1C was drawn on the axial slice above the anterior commissure around the purple stripe. The AF-A was defined as ROI 1A and 1B not 1C, the AF-L was defined as ROI 1A and 1C.

The cingulum bundle cingulate part (CBC): ROIs 2A and 2B were drawn on two coronal slices near the precentral and postcentral gyrus, around the green structures near the midline. The CBC was defined as ROI 2A and 2B.

The uncinate fasciculus (UF), inferior longitudinal fasciculus (ILF) and inferior frontal-occipital fasciculus (IFOF): The ROI 3A and 3B were drawn on the coronal slice of the anterior commissure around the green structure of frontal lobe and temporal lobe respectively. The ROI 3C was drawn on the coronal slice of which the extreme capsule is most concentrated. The ROI 3D was drawn on the coronal slice after the splenium of corpus callosum. The UF was defined as ROI 3A and 3B, the ILF was defined as ROI 3C and 3D not 3A, the IFOF was defined as ROI 3A and 3C and 3D.


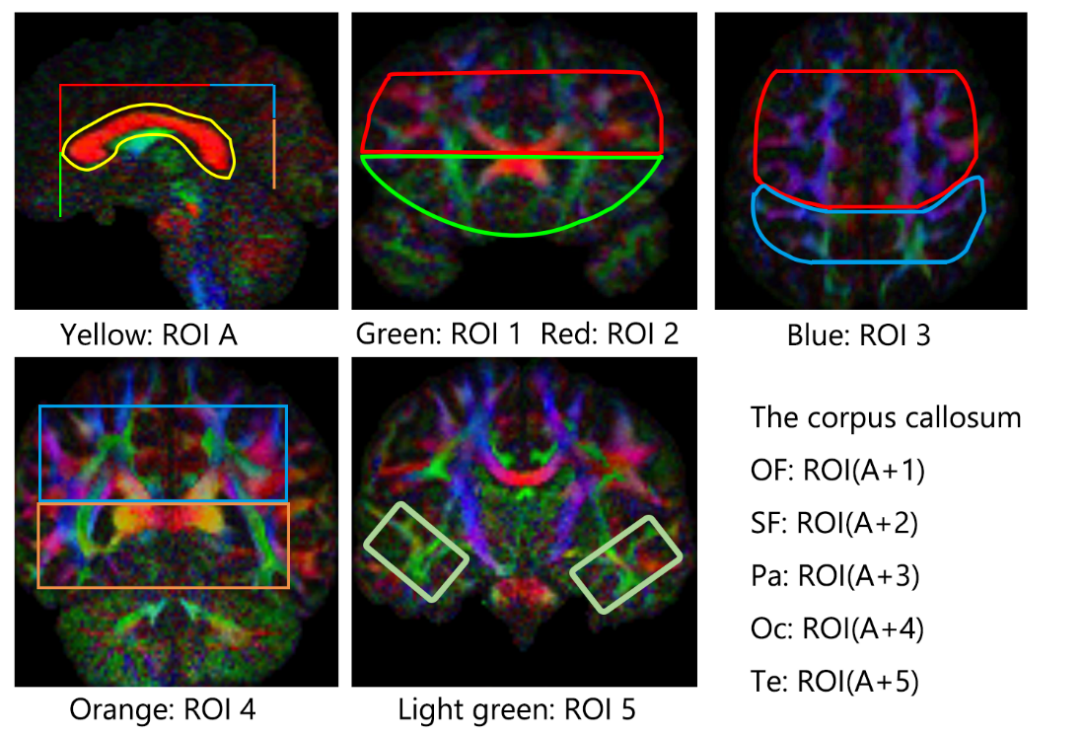


**Supplementary Fig. 3.** The ROIs for the corpus callosum (CC).

ROI A for the CC was drawn on the middle sagittal slice around the red structure, the other ROIs was drawn in the orbital frontal area, superior frontal area, parietal lobe, occipital lobe and temporal lobe perpendicular to ROI A.

1. **Measurements of major fibers in tractography (Table 1)**

| Tracts | AF-A | AF-L | CGC | UF | ILF | IFOF | CC-AF | CC-SF | CC-Pa | CC-Oc | CC-Te |
| --- | --- | --- | --- | --- | --- | --- | --- | --- | --- | --- | --- |
| mean NoF | 1569 | 1150 | 1229 | 956 | 3378 | 1802 | 1592 | 9260 | 2606 | 2500 | 982 |
| NoF AI | -0.363 | 0.312 | 0.109 | -0.201 | 0.061 | -0.028 | - | - | - | - | - |
| mean FA | 0.379 | 0.409 | 0.355 | 0.313 | 0.372 | 0.398 | 0.393 | 0.429 | 0.432 | 0.467 | 0.402 |
| FA AI | -0.041 | 0.003 | 0.029 | -0.031 | -0.036 | -0.013 | - | - | - | - | - |
| FA-Age(r)* | -0.023 | -0.022 | 0.041 | -0.038 | 0.037 | 0.019 | -0.002 | -0.055 | 0.020 | 0.089 | -0.020 |
| mean MD | 0.573 | 0.561 | 0.599 | 0.627 | 0.598 | 0.589 | 0.589 | 0.586 | 0.577 | 0.596 | 0.615 |
| mean AD | 0.813 | 0.823 | 0.832 | 0.838 | 0.848 | 0.858 | 0.846 | 0.877 | 0.864 | 0.920 | 0.885 |
| mean RD | 0.453 | 0.430 | 0.482 | 0.522 | 0.473 | 0.454 | 0.461 | 0.440 | 0.433 | 0.434 | 0.481 |
| mean QA | 3.770 | 4.252 | 3.429 | 3.456 | 3.996 | 4.343 | 4.005 |  | 4.786 | 5.500 | 4.582 |

Mean value of association fibers: (L+R)/2

The asymmetry index (AI) is defined as: AI$=mean(\frac{L\mathrm{index}-R\mathrm{index}}{0.5\left( L\mathrm{index}+R\mathrm{index} \right)})$

*Pearson correlation, no significant result (p<0.05)

1. **The correlations between the tract-wise diffusion properties with CTh or CVo**


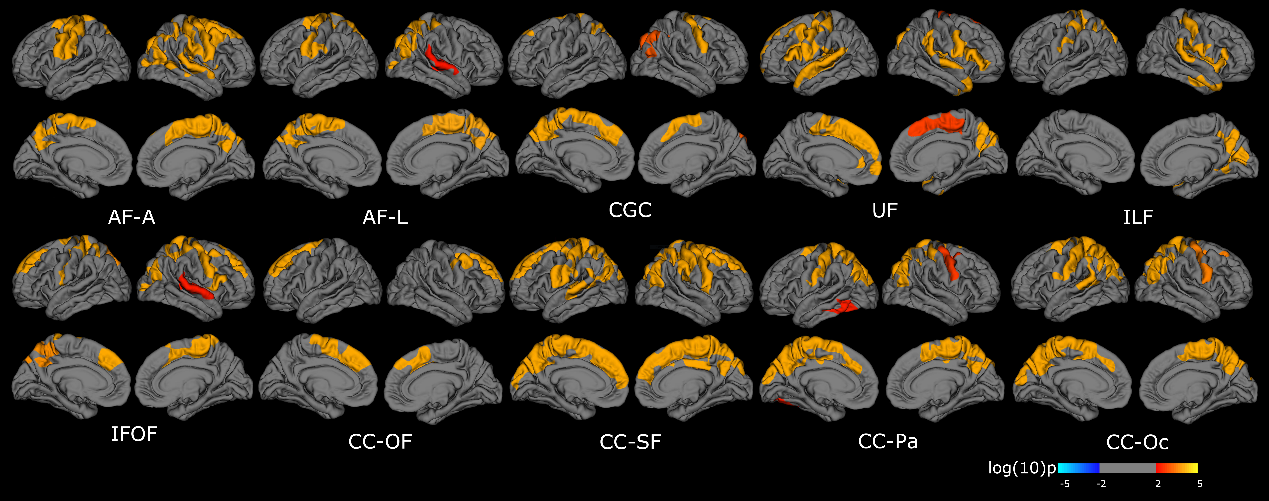


**Supplementary Fig. 4.** The correlation between tract-wise FA values and CTh (after Monte Carol simulation with a threshold of p<0.05)


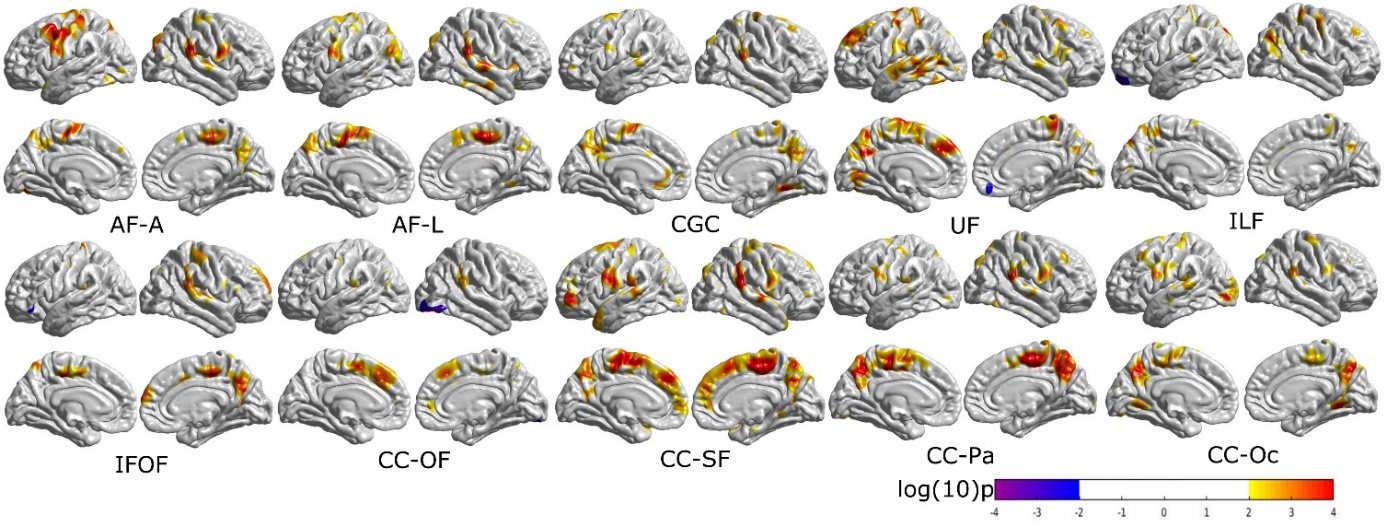


**Supplementary Fig. 5.** The correlation between tract-wise FA value and vertexwise CVo (original p-values with a threshold of p<0.01).


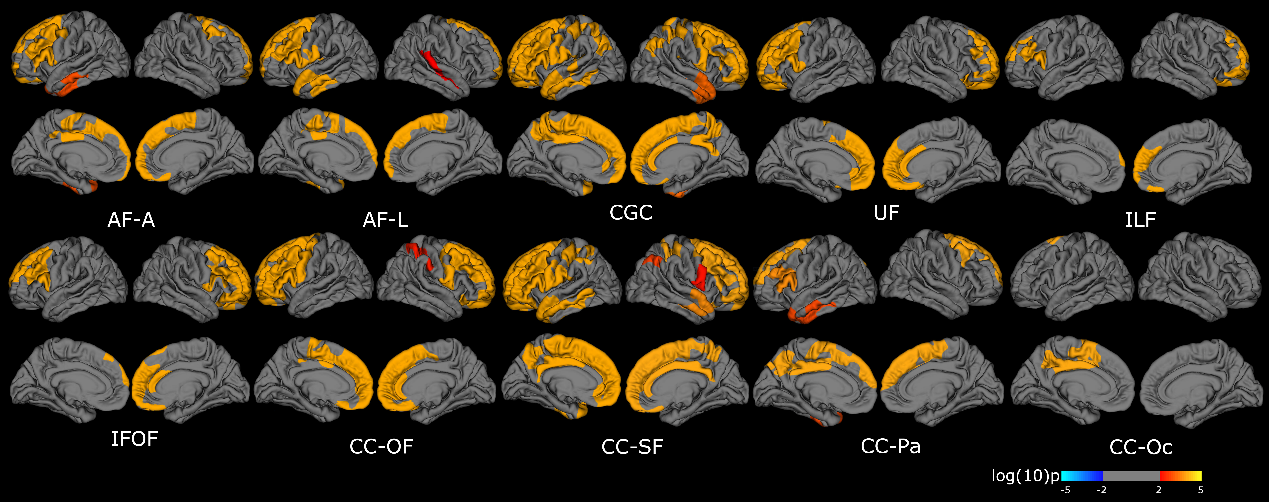


**Supplementary Fig. 6.** The correlation between tract-wise AD values and CTh (after Monte Carol simulation with a threshold of p<0.05)


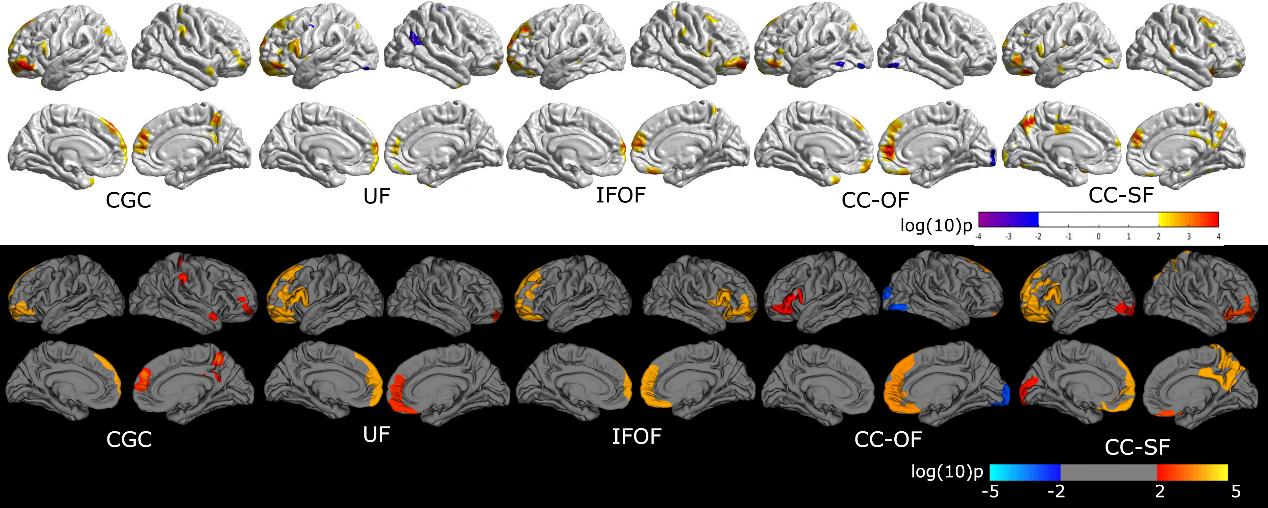


**Supplementary Fig. 7.** The correlation between tract-wise AD and CVo (original p-values with a threshold of p<0.01; and after Monte Carol simulation with a threshold of p<0.05)


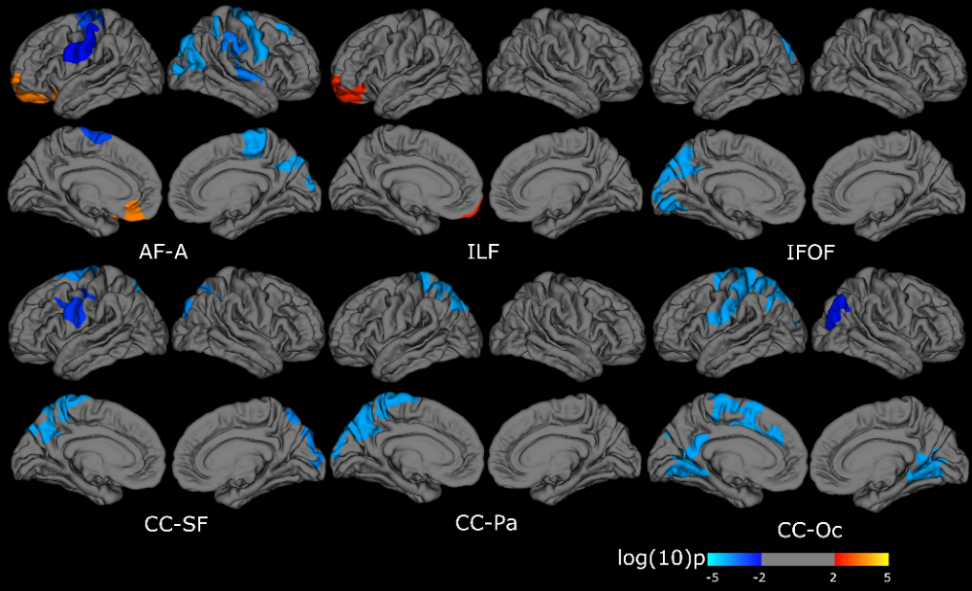


**Supplementary Fig. 8.** The correlation between tract-wise RD and CTh (after Monte Carol simulation with a threshold of p<0.05)


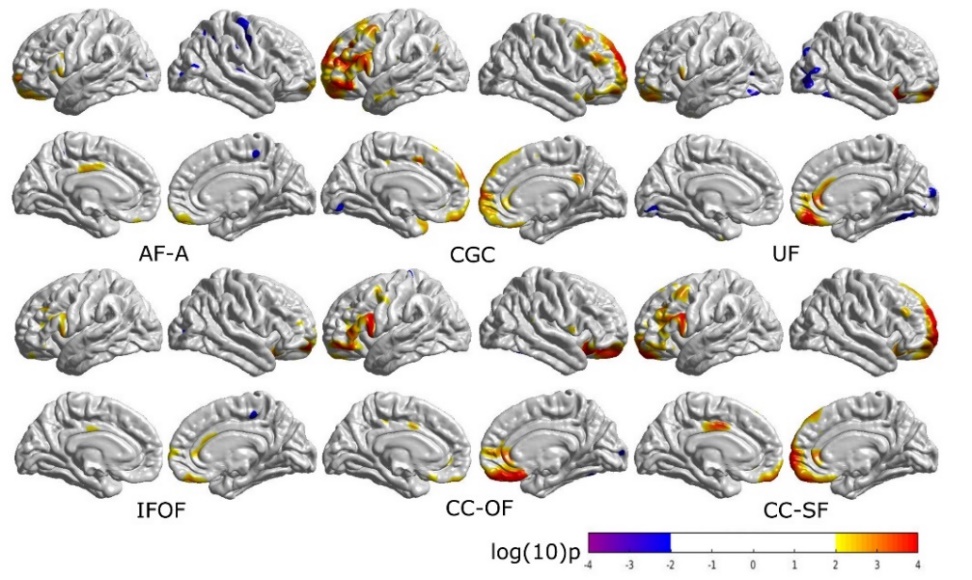


**Supplementary Fig. 9.** The correlation between tract-wise and MD CTh (original p-values with a threshold of p<0.01)

(The CCTemp is not shown because no significant correlation between its diffusion properties of with cortical morphology indices was found.)
